# Role of cholesterol 24S-hydroxylase gene polymorphism (rs754203) in primary open angle glaucoma

**Published:** 2011-02-26

**Authors:** Georg Mossböck, Martin Weger, Christoph Faschinger, Otto Schmut, Wilfried Renner, Andreas Wedrich, Christina Zimmermann, Yosuf El-Shabrawi

**Affiliations:** 1 Department of Ophthalmology, Medical University of Graz, Graz, Austria; 2 Clinical Institute of Medical and Chemical Laboratory Diagnostics, Medical University of Graz, Graz, Austria

## Abstract

**Purpose:**

The enzyme cholesterol 24S-hydroxylase (Cyp46A1) is responsible for the conversion of cholesterol to its more polar metabolite 24S-hydroxycholesterol, thereby enabling the intracerebral elimination of cholesterol. An intronic single nucleotide polymorphism in the gene *CYP46A1* (IVS2 −150 T>C; rs754203) has recently been associated with primary open angle glaucoma (POAG). This association, however, lacks confirmation in other studies. The purpose of the present study was to investigate a hypothesized association between rs754203 and the presence of POAG in a Central European population of Caucasian descent.

**Methods:**

The present institutional study comprised a total of 581 unrelated subjects: 330 patients with POAG, and 251 control subjects. Main outcome measures are genotype distributions and allelic frequencies determined by polymerase chain reaction.

**Results:**

No significant differences in either genotype distribution or allelic frequencies were found between patients with POAG and control subjects (p>0.05). The presence of the rs754203 T-allele was associated with a nonsignificant odds ratio of 0.81 (95% CI: 0.63–1.04; p=0.11) for POAG.

**Conclusions:**

Our data suggest that the rs754203 polymorphism itself is unlikely a genetic risk factor for POAG in Caucasian individuals.

## Introduction

The glaucomas are the most frequent cause of irreversible blindness worldwide [1]. They are defined as progressive optic neuropathies with apoptotic retinal ganglion cell death leading to cupping of the optic nerve with typical visual field defects. Although much effort has been made, the exact pathomechanisms are still elusive [2]. Noteworthy, a positive family history for glaucoma is known to increase the individual risk substantially, albeit most of glaucoma cases are likely to be multifactorial and presumably polygenic with a combination of distinct genetic variants exerting small effects [3-6].

Some debate has been evoked by reports that cholesterol lowering statins may decrease the risk of developing and progression of glaucoma [7-9]. As cholesterol can not cross the blood-brain barrier in significant amounts it has to be synthesized de novo in the central nervous system [10]. For elimination of cholesterol from the central nervous system, it has to be converted into its more polar metabolite 24S-hydroxycholesterol, which is catalyzed by the enzyme cholesterol 24S-hydroxylase (Cyp46A1) [11]. Cyp46A1 is a member of the cytochrome P-450 family and has also been detected in rodent and bovine retinal ganglion cells, indicating a physiologic role in the cholesterol metabolism of the mammalian retina [12,13].

It can be speculated that an altered function of Cyp46A1 may either lead to increased intracerebral cholesterol concentration or accumulation of 24S-hydroxycholesterol. In a rodent glaucoma model hypercholesterolemia has been shown to cause oxidative damage of the retina via induction of nitric oxide synthase 2 [14]. Furthermore, in an in vitro study 24S-hydroxycholesterol has been found to possess neurotoxic properties in physiologic concentration range [15]. Interestingly, in a recent study a positive association between an intronic single nucleotide polymorphism (SNP) in the gene of *Cyp46A1* (IVS2 –150 T>C; rs754203) and POAG was reported in a French population [16]. This association, however, lacks confirmation in other studies.

Our study was thus set to investigate a hypothesized association between rs754203 of *CYP46A1* and POAG.

## Methods

In the present institutional, retrospective case-control study we investigated a total of 581 unrelated Caucasian subjects comprising 330 patients with POAG, and 251 control subjects. All participants were of Caucasian origin, living in the same geographical area and were seen at the local Department of Ophthalmology, Medical University of Graz, Austria. The study was approved by the Institutional Review Board of the Medical University of Graz and followed the principles of the Declaration of Helsinki. Prior to enrolment, written informed consent was obtained from all participants.

All patients underwent slit lamp biomicroscopy, testing for best corrected visual acuity, Goldmann applanation tonometry, gonioscopy, and standard automated perimetry (Interzeag Octopus 500, program G2) or – in cases of profoundly decreased visual acuity – Goldmann perimetry. In all patients photographs of the optic disc were taken. Patients with a known diagnosis of Alzheimer disease were excluded from the study.

POAG was defined by an intraocular pressure (IOP) before initiation of a pressure-lowering therapy of at least 21 mmHg, an open anterior chamber angle, optic disk changes characteristic for glaucoma (notching, thinning of the neuroretinal rim, or increased cup/disc ratio in relation to the optic disc size), visual field defects characteristic for glaucoma (inferior or superior arcuate scotoma, nasal step, or paracentral scotoma) and absence of conditions leading to secondary glaucoma. POAG patients were classified according to their mean defect (MD) on standard automated perimetry using an adapted classification of Hoddap, Parrish, and Anderson [17].

The control group consisted of 251 unrelated patients with no morphological or functional damage indicative for primary or secondary open angle or angle closure glaucoma. Control subjects were admitted to our department for cataract surgery. All participants were Caucasians from the same geographic area (Southern Austria).

### Genotype determination

Venous blood was collected in 5 ml EDTA tubes. DNA was isolated form peripheral lymphocytes using QIAamp DNA blood mini-kit (Qiagen, Venlo, Netherlands) following the manufactures protocol and stored at −20 °C. Genotype determination was performed using high-resolution melting curve analysis on the LightCycler® 480 PCR system (Roche Diagnostics AG, Risch, Switzerland). The samples were amplified in duplicate 20 μl reactions using the Light Cycler 480 High Resolution Melting Master kit (Roche Diagnostics, Wien, Austria) and analyzed on a LC480 instrument I (Roche Diagnostics GmbH, Mannheim, Germany). The final reaction mix contained 1× Master Mix, 3 mM MgCl_2_, 4 μM forward and reverse primer and 50 ng of genomic DNA. For PCR the following cycling conditions were chosen: one cycle of 95 °C for 10 min followed by 45 cycles of 95 °C for 10 s, 60 °C for 15 s, and 72 °C for 20 s. The amplicons were then denaturated at 95 °C for 1 min, cooled down to 40 °C for 1 min, and then melted from 65 °C to 95 °C with 25 signal acquisitions per degree. To detect sequence variations the Gene Scanning Software v1.5 (Roche Diagnostics GmbH) was used. Using the Auto Group mode samples were automatically grouped because of their melting curves.

### Statistical analysis

Descriptive statistics were used to calculate frequencies and percentages of discrete variables. Continuous data are given as mean±standard deviation (SD). Means were compared using Mann–Whitney test. Proportions of groups were compared by the χ^2^ test. Odds ratio (OR) and 95% confidence interval (95%CI) were calculated by logistic regression. The criterion for statistical significance was p≤0.05. Hardy–Weinberg equilibrium has been calculated using HW Diagnostics-Version 1.beta (Fox Chase Cancer Center, Philadelphia, PA). Statistical analysis was done using the SPSS statistical package (SPSS, version 17.0, Chicago, IL). Power calculation was done using PS Power and Sample Size Calculation software version 2.1.30 [18].

## Results

Our study included 330 patients with POAG (196 female and 134 male), and 251 control subjects (126 female and 125 male). The mean age of patients with POAG was 73.5±10.0 years, and 74.2±7.2 years in control subjects, respectively. 59 (17.9%) patients had early (MD above −6dB), 87 (26.4%) had moderate (MD between −6 and −12 dB), and 127 (38.5%) had severe (MD below −12dB) defect.

No significant differences in either genotype distribution or allelic frequencies of rs754203 were found between patients with POAG and control subjects (Table 1). Presence of the rs754203 C-allele was associated with a nonsignificant odds ratio of 1.23 (95% CI: 0.96–1.58; p=0.11) for POAG. Furthermore, no significant differences in either genotype distribution or allelic frequencies of rs754203 were found between patients with early, moderate or severe defect and control subjects (Table 2).

**Table 1:** Genotype distribution and allele frequency of the *CYP46A1* IVS2 –150 C>T polymorphism (rs754203) in patients with primary open angle glaucoma (POAG).

**Genotype**	**Patients with POAG (n=330)**	**Control subjects (n=251)**	**Significance p-value**
rs754203 TT	136 (41.2)	120 (47.8)	0.20
TC	161 (48.8)	112 (44.6)	
CC	33 (10.0)	19 (7.6)	
rs754203 C-allele frequency	0.344	0.299	0.10

Numbers for genotypes are n (%).

**Table 2:** Genotype distribution and allele frequency of the *CYP46A1* IVS2 –150 C>T polymorphism (rs754203) in patients with primary open angle glaucoma (POAG) with early (mean defect [MD] above −6 dB), moderate (MD between −6 and −12 dB) and severe (MD below −12dB) defect.

**Genotype**	**Patients with early defect (n=59)**	**Patients with moderate defect (n=87)**	**Patients with severe defect (n=127)**	**Control subjects (n=251)**	**Significance p-value**
rs754203 TT	26 (44.1)	34 (39.1)	52 (40.9)	120 (47.8)	0.60; 0.16; 0.21*
TC	26 (44.1)	43 (49.4)	63 (49.6)	112 (44.6)	
CC	7 (11.8)	10 (11.5)	12 (9.4)	19 (7.6)	
rs754203 C-allele frequency	0.339	0.362	0.343	0.299	0.39; 0.12; 0.22

Numbers for genotypes are n (%); *=early, moderate, severe.

The present study had a statistical power of 0.80 to detect an odds ratio of 1.42 for the rs754203 C-allele in patients with POAG.

The observed genotype distributions did not deviate from those predicted by the Hardy–Weinberg equilibrium, and for control subjects were similar to those reported for Caucasian populations [19,20].

## Discussion

Conversion of cholesterol to 24S-hydroxycholesterol, catalyzed by Cyp46A1, is the critical step for the elimination of cholesterol from the central nervous system [10]. Alteration of this enzyme may therefore lead to a dysbalance of the intracerebral cholesterol/24S-hydroxycholesterol homeostasis, which may contribute to neurodegenerative diseases like POAG [14,15]. Indeed, a positive association between an intronic SNP in the gene *CYP46A1* (CYP46A1 IVS2 –150 T>C; rs754203) and POAG has been reported [16].

In the present study genotypes of the rs754203 polymorphism were determined in 330 patients with POAG, and 251 control subjects. Allelic frequencies as well as genotype distributions did not significantly differ between patients with POAG and control subjects. This finding is in contrast to data obtained from 150 POAG patients and 118 control subjects in a French study, in which an OR of 1.26 (95% CI: 1.006–1.574; p<0.05) for the TT genotype was reported [16]. The controversial results may be explained by different sample sizes or varying genotype distributions among different populations.

Interestingly, previous studies linked the rs754203 polymorphism to late onset Alzheimer disease (LOAD), a chronic neurodegenerative disease mostly of the elderly like POAG [19-27]. The results of these studies, however, have been controversial and the risk-bearing allele and genotype has not been the same in the positive studies. Of the positive studies, three studies linked the T-allele to an increased risk for LOAD, while five studies suggested the C-allele as risk-bearing allele [19-27].

Generally, intronic SNPs may affect RNA splicing (i.e., exon skipping, intron retention, or introduction of ectopic or cryptic splice sites) or RNA stability, thereby modifying functionality or synthesis rate of a genes product [28]. Papassotiropoulos and coworkers [16,19] provided evidence that the brain β-amyloid load and the cerebrospinal fluid concentration of β-amyloid are increased in individuals with the rs754203 TT genotype, whereas serum as well as cerebrospinal fluid levels of cholesterol and 24S-hydroxycholesterol appeared to be unaffected by the rs754203 genotype. Interestingly, most studies reported decreased levels of β-amyloid in the cerebrospinal fluid of patients with LOAD and likewise, Yoneda and coworkers [29,30] found decreased levels of β-amyloid in the vitreous fluid of patients with glaucoma. As mentioned above, an unambiguous genetic impact for rs754203 has not yet been established indicating that it may be in linkage disequilibrium with a causative locus.

In conclusion, in the present study no statistically significant difference in the genotype and allele distribution of the rs754203 polymorphism was found between patients with POAG and control subjects, suggesting that this polymorphism itself is unlikely a major risk-factor for POAG in a Caucasian population. Further studies are clearly warranted to elucidate the functionality of this polymorphism and its role in POAG.
